# Rapid pain response following intra-articular steroid injections in rheumatoid arthritis finger joints: a prospective study with daily patient self-assessment

**DOI:** 10.1007/s00296-026-06257-3

**Published:** 2026-07-24

**Authors:** Marie Kebke, Emmy Adolfsson, Joakim Strömberg, Mats Dehlin

**Affiliations:** 1 Department of Rheumatology, Södra Älvsborgs Sjukhus, Borås, Sweden; 2 University of Gothenburg, Gothenburg, Sweden; 3 Department of Hand Surgery, Sahlgrenska University Hospital and Institute of Clinical Sciences, Sahlgrenska Academy, Gothenburg, Sweden; 4 Department of Rheumatology, Sahlgrenska University Hospital, Grona Straket 12, Gothenburg, 413 45 Sweden

**Keywords:** Rheumatoid arthritis, Intra-articular injections, Methylprednisolone, Metacarpophalangeal joint, Finger joint, Pain, Range of motion, Treatment outcome, Prospective studies

## Abstract

Intraarticular corticosteroid injections (ISA) are frequently used in rheumatoid arthritis (RA). In spite of this there are few studies on ISA in finger joints in RA. We set out to identify patient-experienced effect of ISA in RA patients regarding pain and mobility of injected arthritic joints, predictors thereof, time span to symptom relief and effect duration. RA patients, ≥ 18 years, were asked to participate just after having received an ISA, with methylprednisolone (MP), in a metacarpophalangeal (MCP) or proximal interphalangeal (PIP) joint, due to arthritis. Patient characteristics, degree of arthritis, CRP and ESR were collected at inclusion. Pain and mobility in the injected joint were reported by the patient on day of inclusion and the following 14 days. Primary outcome was defined as Numerical Rating Scale (NRS) pain ≤ 2 (NRS-scale 0–10) in the injected joint at day 14. Secondary outcomes were NRS mobility, time span and predictors of fulfilling the primary outcome and effect duration. Three-quarters of RA patients responded to ISA regarding pain, most within four days. Mobility improved in two-thirds after 14 days. PIP joints responded better than MCP joints. The only significant predictor was injected MP volume. Among responders, effect persisted for at least three months. ISA is an effective therapy with 75% of RA patients responding over 14 days. PIP joints tend to respond better than MCP joints and the only other predictor for good outcome is volume MP injected, where response increased with volume.

**Supplementary Information:**

The online version contains supplementary material available at 10.1007/s00296-026-06257-3.

## Background

The prognosis of rheumatoid arthritis (RA), the symmetrical small joint arthritic disease engaging hands and feet, has improved dramatically over the last decades due to improved anti-rheumatic treatment. The use of corticosteroids has decreased due to this but they are still quite frequently used, especially to manage a flare of disease. It is preferable to administer corticosteroids intraarticularly (i.a.) when possible, to lessen side effects. In spite of this therapeutic option being available for many years there are few studies on i.a. corticosteroid injections (ISA) in fingers regarding effect on pain, mobility and predictors thereof in RA. These previous studies focus on comparison of routes of administration of steroids, dosages and substances and not on Patient Reported Outcome Measures (PROMs) or predictors of effect [1–7].

We set out to identify patient-experienced effect of ISA in patients with RA regarding pain and mobility of the injected arthritic joint, including the time span to symptom relief. Furthermore, we wanted to identify predictors of good patient-experienced effect of ISA and the duration of the effect.

## Methods

### Patient population

Patients diagnosed with RA by a rheumatologist, aged ≥ 18 years, were asked to participate just after having received an ISA, with methylprednisolone (MP), in a metacarpophalangeal (MCP) or proximal interphalangeal (PIP) joint, due to arthritis, at the Dept of Rheumatology at Sahlgrenska University Hospital, Sweden. Criteria for inclusion were age ≥ 18 years, have a diagnosis of RA set by a rheumatologist, have received an ISA, with MP, in a MCP or PIP joint, due to clinical arthritis at day of inclusion. Patients were excluded if they were not able to read and write Swedish. Patients were included from October 2021 through March 2024.

### Data collection

The following variables were collected at the day of injection: age, sex, smoking (never, former, active), disease duration, seropositivity (defined as presence of rheumatoid factor or anti-citrullinated protein antibodies (ACPA)), erosivity (erosions on hand or feet radiographs), current anti-rheumatic treatment, C-reactive protein (CRP) and erythrocyte sedimentation rate (ESR). Degree of arthritis (mild, moderate, severe) and joint swelling (mild, moderate, severe) were estimated by the physician administering ISA. With numerical rating scales (NRS) from 0 to 10 on global health, pain and mobility of the injected joint were identified at injection day where 0 represents the best outcome and 10 the worst. Volume of MP, in the concentration 40 mg/mL, injected in the studied joint and total amount administered at injection day was recorded. Years working as a rheumatologist was recorded as a proxy for experience in administering ISA. Pain and mobility were then reported daily on a NRS scale for the following 14 days by the patient. A follow-up questionnaire was sent after 3 and 6 months where patients reported pain and mobility in the injected joint on a NRS scale. Possible side effects were collected during follow-up.

### Outcome

Primary outcome was response to ISA defined as NRS pain ≤ 2 in the injected joint at day 14. Secondary outcomes were NRS mobility ≤ 2 in the injected joint at day 14, predictors of fulfilling the primary outcome and effect duration defined as share of responders who reported NRS pain ≤ 2 in the injected joint after 3 and 6 months respectively.

### Ethics

All participants gave written informed consent. The study was approved by the Ethical Review Board of Gothenburg, Sweden (22020 − 05803) 9. of December 2020.

### Statistics

Baseline features were described as count (percent) of categorical data and as mean (standard deviation (SD)) of continuous data. T-test or chi-squared tests (Fisher exact test) were used for comparisons. All data management and analyses were performed in SAS 9.4.

## Results

The 49 patients included in the study had a mean (SD) age of 58 (14) years, the majority were women, 41 (84%), the mean (SD) disease duration was 14 (11) years. The majority, 84%, were seropositive and half had an erosive disease and the vast majority, 46 out of 49 (94%) were on DMARDs, see Table 1.

**Table 1:** Baseline characteristics in total and stratified by response, defined as NRS pain ≤ 2, at day 14

		Total, *n* = 49	Responder, *n* = 37	Non-responder, *n* = 12	*p*-value
Age, mean (SD)		58 (14)	57 (13)	62 (17)	0.3
Sex, female, n (%)		41 (84)	30 (81)	11 (92)	0.4
Smoking, n (%)					0.6*
Never		26 (53)	20 (54)	5 (42)	
Former		21 (43)	15 (39)	6 (50)	
Active		2 (4)	2 (5)	0 (0)	
Seropositive, n (%)		41 (84)	30 (81)	11 (92)	0.7*
Erosive, n (%)		25 (51)	21 (55)	4 (33)	0.3*
Disease duration, years, mean (SD)		14 (11)	15 (11)	11 (9)	0.2
DMARD^a^, n (%)		46 (94)	35 (95)	11 (92)	1*
cs^b^ DMARD		37 (76)	29 (78)	8 (67)	0.5*
b^c^/ts^d^ DMARD		24 (49)	21 (57)	3 (25)	0.1*
b/ts DMARD + csDMARD		16 (33)	15 (41)	1 (8)	0.07*
Prednisolone		7 (14)	4 (11)	3 (25)	0.3*
Global health at injection day, mean (SD) NRS 0–10		5.4 (2.4)	5.2 (2.5)	6.3 (1.8)	0.1
Degree of arthritis					0.6*
Mild, n (%)		8 (16)	5 (13)	3 (25)	
Moderate, n (%)		31 (63)	24 (65)	7 (58)	
Severe, n (%)		10 (20)	8 (21)	2 (17)	
Degree of swelling (1–3)					
Mild, n (%)		9	6 (16)	3 (25)	0.6*
Moderate, n (%)		27	20 (54)	7 (58)	
Severe, n (%)		8	7 (18)	1 (8)	
CRP, mean (SD)		7 (8)	6 (6)	9 (13)	0.4
ESR, mean (SD)		23 (17)	23 (17)	26 (19)	0.6
Years as rheumatologist, years, mean (SD)		15 (6)	16 (6)	13 (6)	0.06
Years as rheumatologist, years, median below, n (%)		29 (59)	19 (51)	10 (83)	0.09*
Years as rheumatologist, median above, n (%)		20 (41)	18 (49)	2 (17)	
Injected joint, n (%)					
MCP**		25 (51)	15 (60)	10 (40)	0.02*
PIP***		24 (49)	22 (92)	2 (8)	
Volume of MP**** injected in the joint, ml, mean (SD),		0.32 (0.1)	0.34 (0.1)	0.26 (0.09)	0.039
Volume of MP**** injected in total, ml, mean (SD)		0.8 (0.6)	0.79 (0.6)	0.84 (0.7)	0.8

* Fisher exact test, **MCP = MetaCarpoPhalangeal joint, ***PIP = Proximal Interphalangeal joint, ****MP = methylprednisolone 40 mg/mL, a= Disease-Modifying AntiRheumatic Drug, b = Conventional Synthetic Disease-Modifying Anti-Rheumatic Drug, c=Biologic Disease-Modifying Antirheumatic Drug, d= Targeted Synthetic Disease-Modifying Anti-Rheumatic Drug

**Fig. 1:**
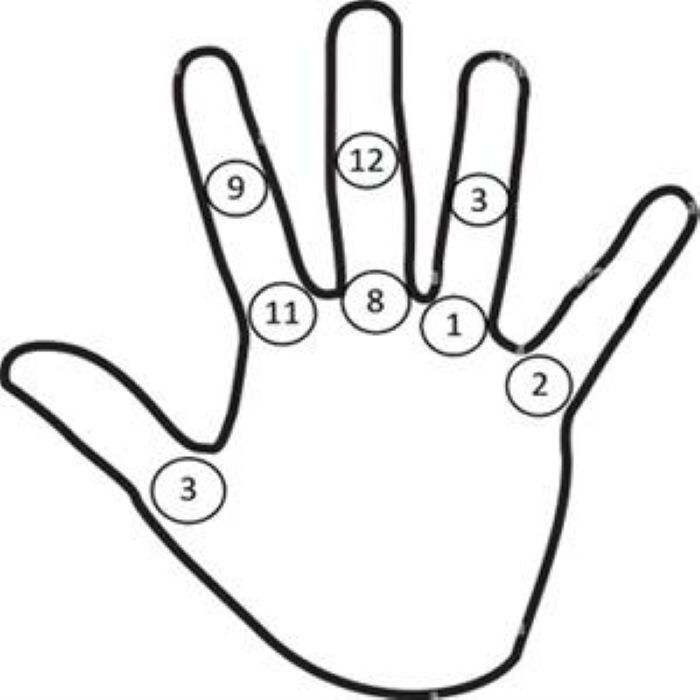
Distribution of corticosteroid injections by joint location (MCP and PIP joints). The numbers within the circles represent the total number of injections performed in each specific joint with left and right hand summed up

PIP 3 was the most frequent injected joint, see Fig. 1 for more details on this.

**Fig. 2:**
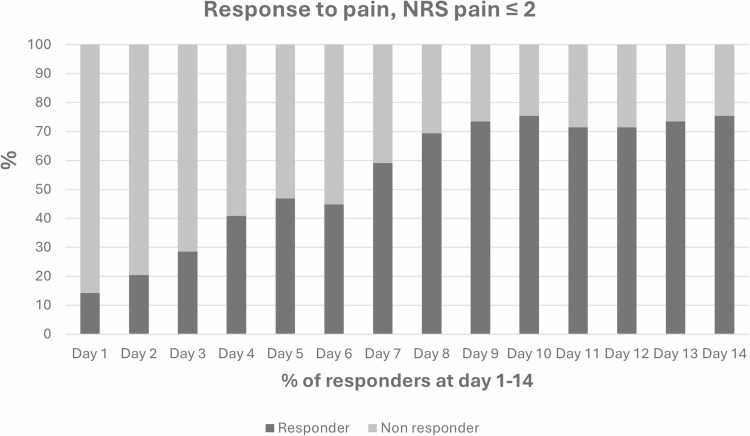
Primary outcome, treatment response, responder defined as Pain NRS ≤ 2, from day 1–14

On day 14, 37 (76%) patients reported NRS pain ≤ 2 in the injected joint, the majority reporting this already after 4 days, see  Fig. 2.

Regarding mobility, 31 (63%) patients reported NRS ≤ 2 in the injected joint on day 14. Comparing responders and non-responders to pain no significant differences were found in demographics, disease characteristics, smoking status and degree of inflammation in the joint / blood, Table 1. The only significant predictor of response was a higher mean (SD) volume of MP injected, 0.34 (0.1) versus 0.26 (0.09) ml, respectively (*p* = 0.039). There was a trend towards better effect of ISA related to experience defined as years worked as a rheumatologist, *p* = 0.06. PIP joints displayed significantly better response to ISA compared to MCP joints, *p* = 0.02 (Table 1).

PROMs showed prolonged effect of ISA, after 90 days following the injection, 24 (65%) of the responders reported NRS pain ≤ 2 in the injected joint and at 180 days this had decreased to 18 (49%). No significant side effects were reported during the study.

## Discussion

In this prospective observational study, we found that 3/4 of RA patients responded to ISA and the majority did this within four days. The only significant predictor was volume MP injected. PIP joints responded better than MCP joints. The majority of responders reported PROMs supporting the effect lasted for at least 3 months.

In the study by Helliwell et al. from 1997 [2], the effects of ISA, with triamcinolone hexacetonide (TH), in MCP-3 on articular stiffness, strength, joint range of movement, and VAS pain and stiffness, was analyzed in 15 patients / 17 joints with inflammatory arthritis. After seven days 12 out of 17 (71%) joints had improved, mean VAS pain at baseline was 50 and decreased to 12 after one week.

In our study, PIP joints responded better to ISA compared to MCP joints. In the study by Hetland et al. from 2012 [8], short- and long-term efficacy of i.a. betamethasone injections were tested in 160 patients with early RA. Ankles, elbows, knees, shoulders, wrists, MCP and PIP joints were injected during 2 years. After 2 years, no relapse had been seen after first injection in 73.7% of PIP-joints compared to 52.3% of MCP-joints, suggesting a better responsiveness to ISA in PIP-joints.

The majority (65%) of the responders in our study had a duration of effect > 3 months regarding pain and almost half, 49%, after 6 months. In the study by Javala et al. from 1983 [3], the effects on articular circumference in the PIP joints between TH and MP were compared in a total of 120 joints over 24 weeks. After 24 weeks there was a beneficial result in 75.5% of the finger joints in the TH group but in the MP group the corresponding figure was 58%.

The patients in our study reported no significant side effects during the six month follow-up. In the web-based survey by de la Torre-Aboki et al. from 2021 targeting people who had experienced at least two intra-articular injections in various joints, with different substances injected and with different indications, complications were reported by 20% [9]. In a recent review on safety of ISA, Duarte-Montero et al. conclude that most studies report mild and self-limited adverse events with an incidence similar to placebo [10].

There are some limitations to our study. There was no control group to compare the effects with. However, in the preparations for the study we found it unethical to have a placebo group. To add an ultrasound examination to the study would probably have helped to sift out the joints with inflammatory arthritis and it would probably have led to increased effect sizes. In the study we only used self-reported follow-up, clinical follow-up would have added valuable information, but the study was performed during the Covid pandemic and in-person meetings with patients were advised to be kept at a minimum. Another limitation is the relatively small sample size (49 vs. the planned 140 participants), largely due to slow recruitment during the COVID-19 pandemic, which limited the statistical power to reliably identify predictors of treatment response.

There are also some strengths to our study. It adds information on the trajectory of symptom relief after a very common, but less studied, practice in rheumatology. To our knowledge, this is the first study with a day-to-day follow-up after ISA. The study is based on “real-world” data, the patients who were enrolled had come for ISA on their own initiative and were included after the injection.

## Conclusion

To conclude, ISA is an effective therapy with 75% of RA patients responding over 14 days. PIP joints tend to respond better than MCP joints and the only other predictor for good outcome is volume MP injected.

## Supplementary Information

Below is the link to the electronic supplementary material.

Supplementary Material 1

## Declarations

### AI

No part of this report is copied, published elsewhere, or generated by AI. No AI was used for drafting or editing this report.
